# Supplementary material for: Microbiological and Molecular Features Associated with Persistent and Relapsing Staphylococcus aureus Prosthetic Joint Infection
Source: Antibiotics (Basel). 2022 Aug 18;11(8):1119. doi: 10.3390/antibiotics11081119 (PMC9405193; doi:10.3390/antibiotics11081119)
Supplement: Supplementary file 1 [file antibiotics-11-01119-s001.zip › antibiotics-1850997-supplementary.pdf]

Figure S1. Cytotoxicity activity, alpha-haemolysin, biofilm formation and fibronectin binding capacity by *S. aureus* strains included in our study. (A). Haemolysin alpha was evaluated by quantifying *hla* production by using rabbit erythrocytes as the final dilution (DF, dilution factor) of concentrated bacterial supernant where complete hemolysis occurred. **(B).** Biofilm forming abilities of the PJI *Staphylococcus aureus* clinical isolates was assessed by OD_{595} into 96-well microtiter plates for 24 h using crystal violet staining method. **(C).** Abilities of the different *S. aureus* clinical strains to bind to fibronectin. **(D).** Cytotoxicity activity of PJI *Staphylococcus aureus* in MG63 osteoblasts evaluated by quantifying LDH release at 24h post infection. Results were expressed as percentage cytotoxicity as the difference in LDH release between the infected and infected cells**.** The horizontal bars denote the median derived from three independent experiments and the vertical bars denote the interquartile range (IQR). All the *P* values were calculated by using Mann-Whitney U-test.

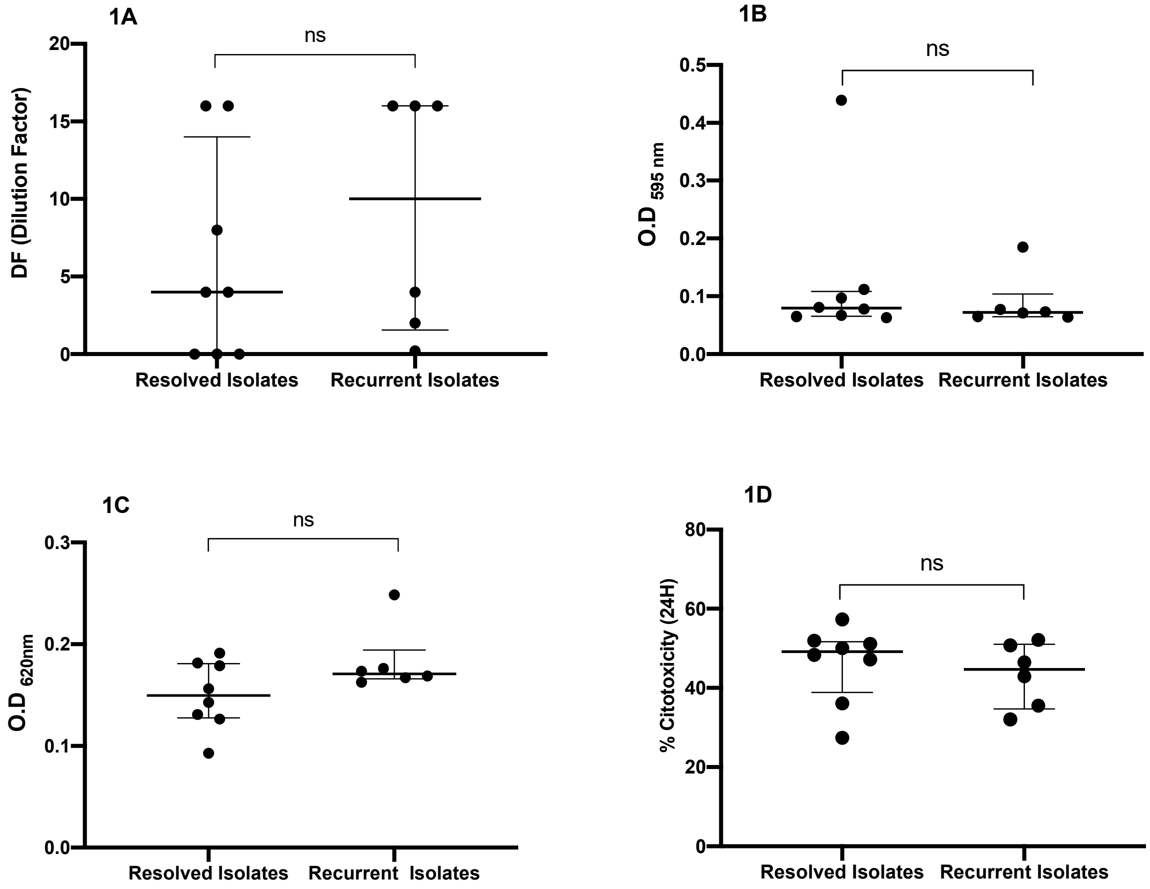

Figure S2. Comparisons in the adhesion, invasion and persistence capacities between initial and second *S. aureus* isolates. Initial isolates: *S. aureus* isolates recovered at initial time of diagnosis prior to surgery; Second isolates: *S. aureus* isolates obtained after or during antimicrobial treatment. Results were expressed as the median and interquartile range. Statistical analysis was performed using Mann-Whitney U-test (ns: no significant).

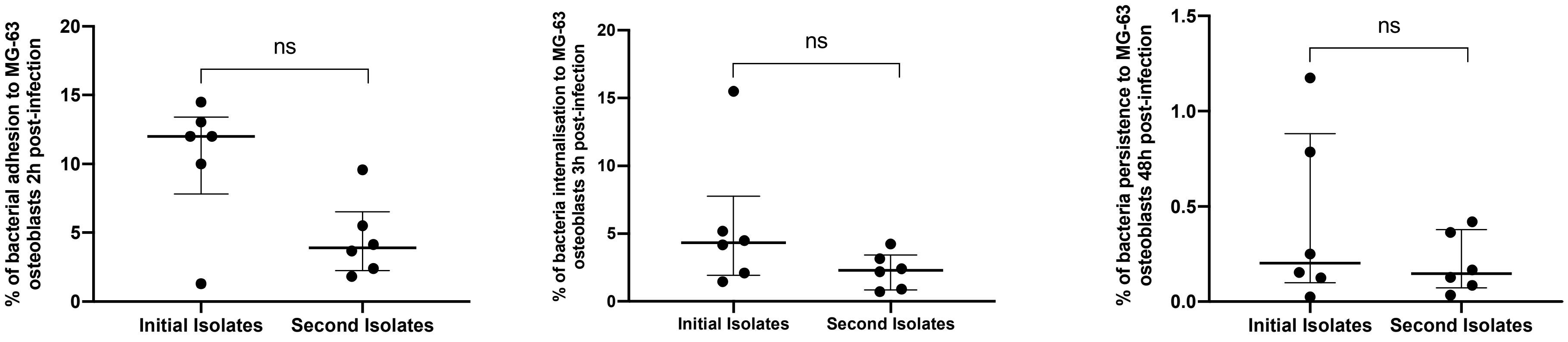

Figure S3. Ability to form biofilm of initial- and second *S. aureus* isolates retrieved from the same patient at the initial time of diagnosis and at the time of persistence or relapsed. Results show the means ± standard deviation (SD). Statistical analyses were performed by using Mann-Whitney U-test (ns: no significant).

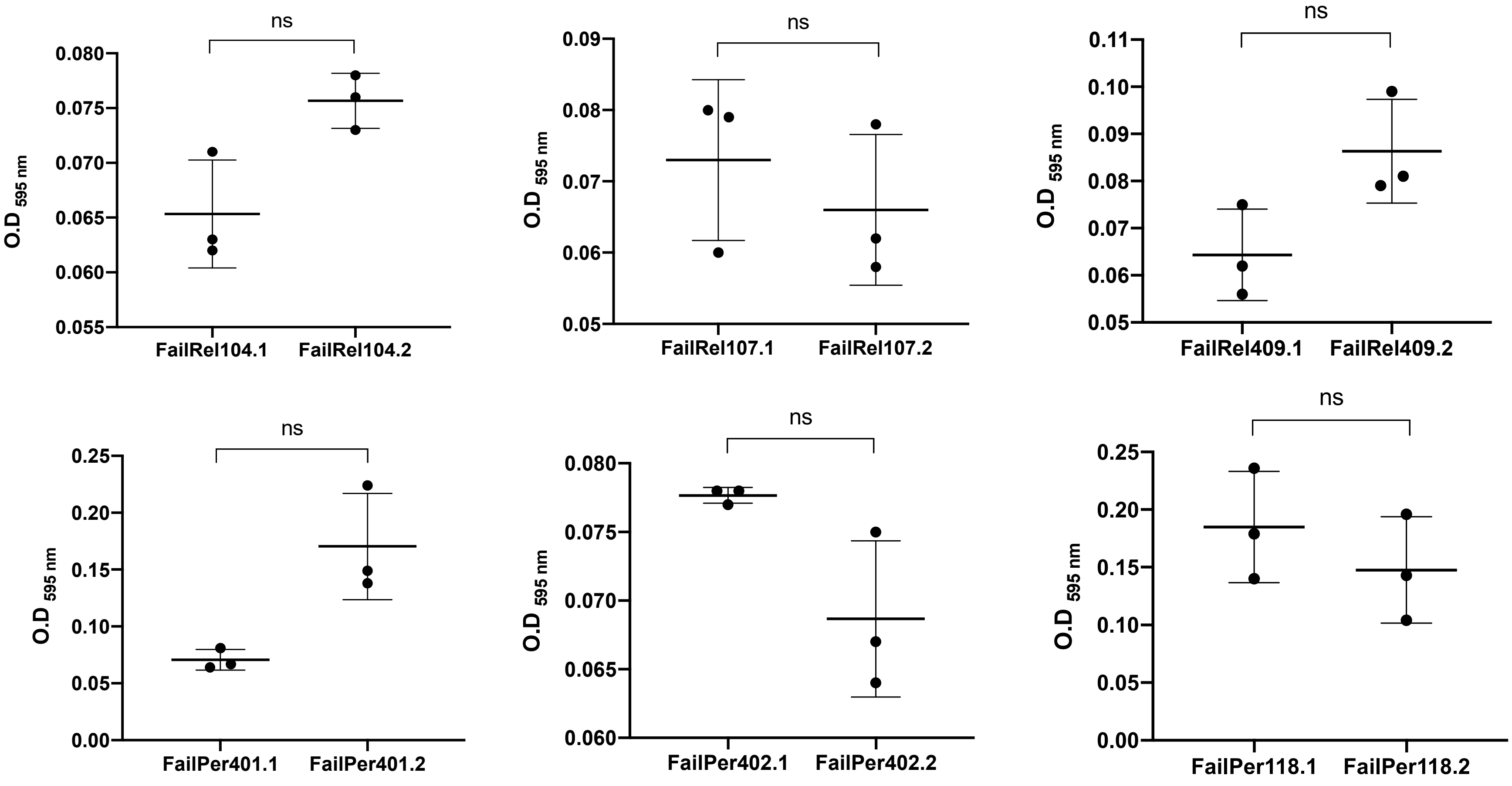

Figure S4. Cytotoxic effect after infection between the different pairs of *S. aureus* grouped into relapse- or persistent- in human osteoblasts. The results are expressed as the percentage of the value of LDH released 24 h post infection and uninfected cells. The horizontal bars denote the means ± SD of three independent experiments. No significant differences (ns) were observed between any pair of recurrent- or persistent/relapsed- *S. aureus* isolate (Mann-Whitney U test).

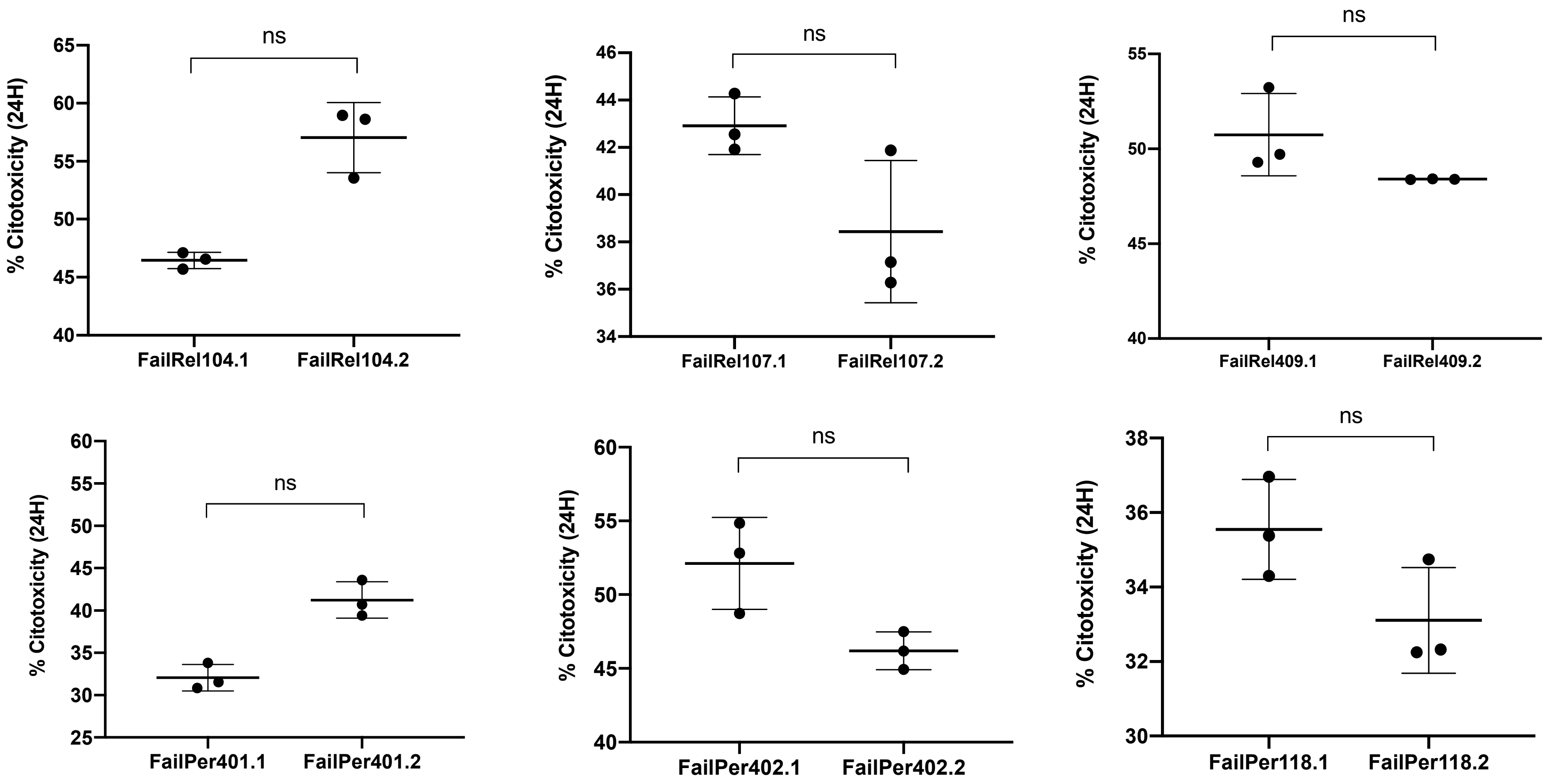

Figure S5. Abilities of *S. aureus* strains to bind to fibronectin. Data are the means ± standard deviation (sd) of three replicate experiments. Comparison of the data using Mann-Whitney showed no significant difference between the initial *S. aureus* strain isolated at time of diagnosis and persistent or relapsed strains (ns: no significant).

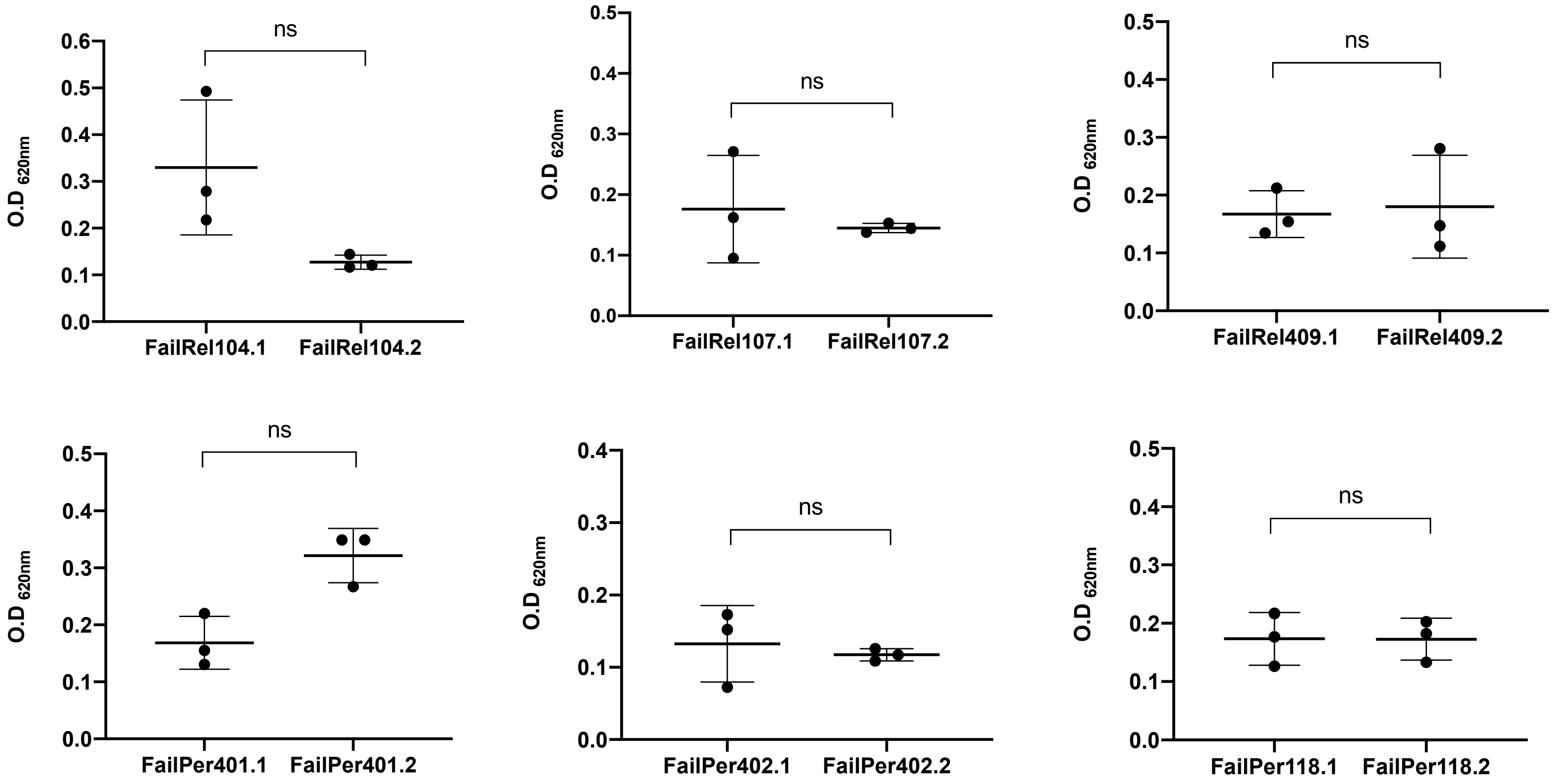

Table S1. Molecular epidemiology of *S. aureus* strains causing PJI and reference strains used for each strain and each bioinformatic analysis.

**Strain** (code)	**CC**	**ST**	***spa type***	**Reference**[a] (annotation)	**Reference** (variants)[b]
FailRel104.1	15	15	-	MSSA476	MSSA476
FailRel104.2	15	15	-	MSSA476	MSSA476
FailRel107.1	398	398	-	SO385	SO385
FailRel107.2	398	398	-	SO385	SO385
FailRel409.1	188	188	-	MSSA476	MSSA476
FailRel409.2	188	188	-	MSSA476	MSSA476
FailPer401.1	45	45	t026	CA-347	CA-347
FailPer401.2	45	45	t026	CA-347	CA-347
FailPer402.1	5	5	-	N315	N315
FailPer402.2	5	5	-	N315	N315
FailPer118.1	5	125	t067	N315	N315
FailPer118.2	5	125	t067	N315	N315
Cure116.1	5	5	-	N315	N315
Cure804.1	10	10	-	N315	N315
Cure806.1	45	45	-	CA-347	N315
Cure111.1	30	30	-	MRSA252	N315
Cure112.1	6	6	-	N315	N315
Cure208.1	509	509	-	N315	N315
Cure807.1	45	45	-	CA-347	N315
Cure808.1	1	852	-	MSSA476	N315

Abbreviations: CC, clonal complex; ST, sequence type.

[a]The CCs y STs of reference strains are as follow: MSSA476 (CC1, ST1, BX571857.1), SO385 (CC398, ST398, AM990992.1), N315 (CC5, ST5, BA000018.3), MRSA252 (CC30, ST30, BX571856.1), CA-347 (CC45, ST45, CP006044.1).

[b]The variant analysis was performed with the reference strain N315 (CC5), since it was the major CC, except for the variant analysis between each pair of strains from the same patient with persistent or relapsing PJI, for which the reference strain most suitable for the CC of each pair of strains was chosen.

Table S2. Virulence genes of all the strains causing the 14 prosthetic joint infections included in this study[1].

Virulence	Gene	All cases (n=14) No. (%)	Persistence/relapse (n=6) No. (%)	Cure (n=8) No. (%)
Proteases	*splA*	6 (42.9)	3 (50.0)	3 (37.5)
	splB	6 (42.9)	3 (50.0)	3 (37.5)
	aur	14 (100.0)	6 (100.0)	8 (100.0)
Enterotoxins	*seg*	8 (57.1)	3 (50.0)	5 (62.5)
	sea	4 (28.6)	1 (16.7)	3 (37.5)
Toxic shock toxin	*tst*	1 (7.1)	0 (0.0)	1 (12.5)
Leukocidins	*lukE*	8 (57.1)	4 (66.7)	4 (50.0)
	lukD	8 (57.1)	4 (66.7)	4 (50.0)
Haemolysins	*hlg*	14 (100.0)	6 (100.0)	8 (100.0)
	hla	14 (100.0)	6 (100.0)	8 (100.0)
Hlb-phages	*scn*	13 (92.9)	5 (83.3)	8 (100.0)
	sak	11 (78.6)	4 (66.7)	7 (87.5)
	chp	10 (71.4)	4 (66.7)	6 (75.0)
MSCRAMMs (microbial surface components recognizing adhesive matrix molecules)	*clfA*	11 (78.6)	5 (83.3)	6 (75.0)
	clfB	10 (71.4)	3 (50.0)	7 (87.5)
	cna	9 (64.3)	3 (50.0)	6 (75.0)
	fnbpA	14 (100.0)	6 (100.0)	8 (100.0)
	fnbpB	12 (85.7)	6 (100.0)	6 (75.0)
	ebpS	14 (100.0)	6 (100.0)	8 (100.0)
	spa	14 (100.0)	6 (100.0)	8 (100.0)
	sdrC	14 (100.0)	6 (100.0)	8 (100.0)
	sdrD	11 (78.6)	6 (100.0)	5 (62.5)
Extracellular proteins	*coA*	14 (100.0)	6 (100.0)	8 (100.0)
	efb	14 (100.0)	6 (100.0)	8 (100.0)
Biofilm formation	*atl*	14 (100.0)	6 (100.0)	8 (100.0)
LPXTG motif	*sasG*	7 (50.0)	3 (50.0)	4 (50.0)
Polysaccharide intracellular adhesin (PIA)	*icaB*	14 (100.0)	6 (100.0)	8 (100.0)
	icaC	14 (100.0)	6 (100.0)	8 (100.0)
	icaD	14 (100.0)	6 (100.0)	8 (100.0)

[1]In relapsing/persistent cases it has only been included the first strain for this analysis.
